# The Effect of Umbilical Cord-Derived Mesenchymal Stem Cells and Secretome on Metabolomic Profiles (C-Peptide, Adiponectin, Fasting Insulin, and Fasting Glucose): A Randomized Controlled Trial

**DOI:** 10.3390/jcm15051707

**Published:** 2026-02-24

**Authors:** Gunawan Dwi Prayitno, Cynthia Retna Sartika, Tono Djuwantono, Andi Wijaya, Raden Muharam, Yudi Mulyana Hidayat, Rima Haifa, Annisah Zahrah, Keri Lestari

**Affiliations:** 1 Doctoral Program of Pharmacy, Faculty of Pharmacy, Universitas Padjadjaran, Jatinangor 45363, Indonesia; gunawan20001@unpad.ac.id; 2 Department of Obstetrics and Gynecology, Gatot Soebroto Army Central Hospital, Central Jakarta City 10410, Indonesia; 3 PT Prodia StemCell Indonesia, DKI Jakarta, Central Jakarta City 10430, Indonesia; c.sartika@gmail.com (C.R.S.); aw.prodia@gmail.com (A.W.); rima.haifa@prostem.co.id (R.H.); 4 Department of Obstetrics and Gynecology, Faculty of Medicine, Universitas Padjadjaran, Jatinangor 45363, Indonesia; tono.djuwantono@unpad.ac.id (T.D.);; 5 Department of Obstetrics and Gynecology, Faculty of Medicine, Universitas Indonesia, Depok 16424, Indonesia; 6 Department of Pharmacology and Clinical Pharmacy, Faculty of Pharmacy, Universitas Padjadjaran, Jatinangor 45363, Indonesia

**Keywords:** PCOS, mesenchymal stem cells, secretome, insulin resistance, adiponectin, epigenetics, inflammation

## Abstract

**Background:** Polycystic ovary syndrome (PCOS) is a prevalent endocrine–metabolic disorder with chronic low-grade inflammation and insulin resistance (IR). Elevated C-peptide, a marker of compensatory hyperinsulinemia and reduced adiponectin, an insulin-sensitizing adipokine, contribute to the metabolic dysregulation observed in PCOS. Human umbilical cord-derived mesenchymal stem cells (UC-MSCs) and their secretome have immunomodulatory properties via paracrine and epigenetic mechanisms, yet longitudinal evidence in PCOS is limited. **Methods:** This randomized controlled trial (RCT) involved 40 women with PCOS (Rotterdam criteria) who were randomly assigned to four treatment groups: (1) metformin 750 mg/day, (2) UC-MSC infusion (0.3 million cells/kg body weight), (3) secretome (nasal drops, 2 mL), and (4) a combination of UC-MSC (0.3 million cells/kg body weight) and secretome (nasal drops, 2 mL). Parameters measured included fasting glucose, fasting insulin, HOMA-IR, C-peptide, and adiponectin at baseline and at months 1, 3, and 6. Analysis was performed using repeated-measures ANOVA or Friedman test, and ROC curves were used to evaluate the predictive value of biomarkers on therapy response. **Results:** All participants completed the 6-months of follow-up. The secretome group demonstrated a significant increase in fasting glucose (month 1: *p* = 0.013; month 3: *p* = 0.007; month 6: *p* = 0.032), as well as an increase in adiponectin in the UC-MSC group (month 6: *p* = 0.016). The combination of UC-MSC and secretome induced early metabolic modulation, characterized by transient reductions in adiponectin at months 1 and 3 (*p* = 0.022 and *p* = 0.013, respectively) and early increases in insulin-related parameters; however, these effects were not sustained at month 6. ROC analysis showed that glucose, insulin, and C-peptide variables had low discriminatory ability (AUC < 0.5), while adiponectin showed a trend of increasing predictive value for improving insulin sensitivity. **Conclusions:** Combination therapy with UC-MSCs and secretome may have potential to improve metabolic profiles through increasing adiponectin and improving insulin sensitivity in PCOS patients, especially in the group with insulin resistance. MSC-based approaches are not only symptomatic but also have the potential to restore ovarian function through immunomodulatory and epigenetic mechanisms.

## 1. Introduction

Polycystic ovary syndrome (PCOS) is the most common endocrine disorder in women of reproductive age, with a global prevalence ranging from 6 to 15% [1,2,3]. The etiology of PCOS is complex, heterogeneous, and incompletely understood, but current evidence suggests the involvement of genetic, endocrine, metabolic, and environmental factors in the clinical manifestations of the syndrome [4]. A recent meta-analysis estimated the prevalence of PCOS at 9.2% (95% CI: 6.8–12.5%), although this figure varies according to the diagnostic criteria used: 5.5% according to the NIH criteria, 11.5% according to the Rotterdam criteria, and 7.15% according to the AES criteria [5]. These differences reflect variations in diagnostic definitions: the Rotterdam criteria define the diagnosis as the presence of at least two of the three main components (hyperandrogenism, oligo-anovulation, polycystic ovaries), the NIH criteria require chronic anovulation and evidence of hyperandrogenism, while the AES criteria emphasize hyperandrogenism accompanied by ovarian dysfunction [6,7].

Insulin resistance (IR) represents a central pathophysiological feature of PCOS and is reported in approximately 40–70% of affected women, particularly among those with obesity [8]. IR and chronic hyperinsulinemia promote low-grade systemic inflammation, which disrupts folliculogenesis, impairs implantation, increases miscarriage risk, and contributes to obstetric complications such as preeclampsia. In parallel, chronic anovulation leads to unopposed estrogen exposure, increasing the risk of endometrial hyperplasia and carcinoma. From a metabolic perspective, women with PCOS have an elevated risk of developing type 2 diabetes mellitus, metabolic syndrome, and cardiovascular disease [9,10,11,12,13].

The mechanisms underlying IR in PCOS extend beyond impaired insulin receptor signaling and involve dysregulation of adipokines and metabolic biomarkers associated with insulin action. Adiponectin, an adipokine with insulin-sensitizing and anti-inflammatory properties, is consistently reduced in PCOS and is inversely correlated with HOMA-IR, supporting its role as a protective metabolic marker [11,14,15]. In contrast, elevated C-peptide reflects compensatory endogenous insulin secretion and persistent hyperinsulinemia, which contributes to both metabolic and reproductive dysfunction in PCOS.

In recent years, metabolomic approaches have become essential tools for understanding the biochemical complexity of PCOS. Metabolomics is a branch of “multi-omics” science that focuses on the characterization of small metabolites (<1 kDa),such as amino acids, fatty acids, lipids, sugars, and citric acid cycle products, that dynamically reflect the body’s physiological and pathological status [16]. Because metabolites are the end products of gene and protein activity, metabolomic analysis provides the closest possible representation of the actual phenotype, potentially revealing specific biochemical dysfunctions in PCOS. The primary goals of metabolomics are to identify metabolic biomarkers for disease diagnosis and prediction, understand the mechanisms of metabolic and inflammatory disorders, evaluate therapeutic responses, and elucidate gene–environment interactions at the molecular level. This approach allows for a comprehensive analysis of changes in metabolite profiles that cannot be explained solely by conventional hormonal or biochemical assays.

Advances in systems biology, including metabolomics, have contributed to a deeper understanding of the biochemical complexity of PCOS. Metabolomic studies have identified characteristic alterations involving oxidative stress-related metabolites (such as malondialdehyde and acylcarnitine), impaired branched-chain amino acid (BCAA) metabolism, and disruption of mitochondrial energy pathways, linking insulin resistance with chronic inflammation and ovarian dysfunction [17,18]. This profile suggests a link between mitochondrial dysfunction, insulin resistance, and systemic inflammation. Furthermore, biomarkers such as C-peptide and adiponectin are increasingly used to reflect metabolic and hormonal dysregulation in PCOS [14,19]. Thus, a metabolomic approach offers the opportunity to understand PCOS holistically and serve as a basis for developing more precise and targeted therapies for metabolic and inflammatory disorders.

Conventional treatments, particularly metformin, primarily target insulin sensitivity but exhibit limited effects on inflammatory and epigenetic pathways implicated in PCOS pathophysiology. Consequently, regenerative approaches using UC-MSCs and their bioactive secretome have emerged as promising therapeutic strategies. The secretome, composed of cytokines, growth factors, bioactive proteins, extracellular vesicles, and microRNAs, mediates many of the paracrine effects of MSCs and has been shown to modulate inflammation, insulin signaling, and tissue repair [20,21].

Preclinical studies demonstrate that UC-MSCs can improve hormonal balance, reduce inflammation, and restore ovarian function in PCOS models. In addition, systematic reviews suggest that MSC-derived secretomes may exert immunomodulatory effects that influence metabolic and reproductive outcomes [20,22]. However, clinical evidence in human PCOS populations remains limited, and the differential effects of UC-MSCs, secretome, and their combination on metabolic biomarkers and reproductive outcomes have not been fully elucidated.

Against this background, this study aimed to evaluate the effect of UC-MSCs and secretome-based therapy on metabolic and adipokine biomarkers associated with insulin resistance and reproductive outcomes in women with PCOS. This approach is expected not only to provide a more comprehensive and sustainable therapeutic alternative but also to establish a scientific basis for the development of future epigenetic-based treatment strategies.

## 2. Materials and Methods

### 2.1. Study Design and Participants

This study was designed as an exploratory, randomized controlled trial (RCT) conducted between 2022 and 2024 at Gatot Soebroto Army Hospital, Central Jakarta City, Indonesia. The study was approved by the institutional ethics committee (approval numbers: 26/VII/KEPK/2022 and 54/III/KEPK/2024) and was conducted in accordance with the Declaration of Helsinki. All participants provided written informed consent prior to enrollment. This study was registered in a public clinical trials registry (ClinicalTrials.gov identifier: NCT05279768). No changes to the study methods were made after trial commencement.

The sample size was calculated using Lemeshow’s formula for comparative clinical studies, with a significance level (α) of 0.05 and statistical power (1 − β) of 90%. Based on the expected variability of metabolic biomarkers and the exploratory design of this pilot randomized controlled trial, a minimum total sample size of 40 participants was determined, with 10 subjects allocated to each intervention group.

### 2.2. Participants and Eligibility Criteria

**Figure 1:**
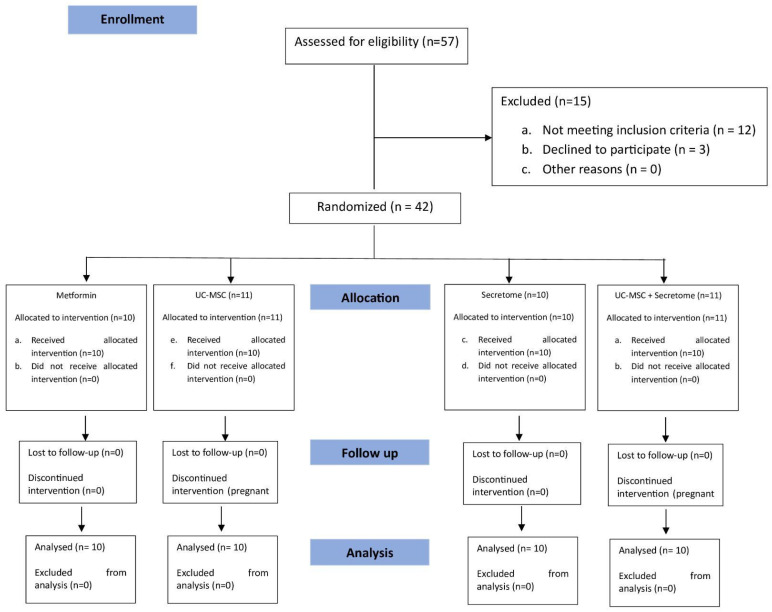
CONSORT flow diagram of participant recruitment, randomization, treatment allocation, and follow-up through 6 months. Different colors indicate different study phases (enrollment, allocation, follow-up, and analysis).

A total of 40 women aged 20–40 years with a diagnosis of polycystic ovary syndrome (PCOS) were enrolled (Figure 1). PCOS was diagnosed according to the Rotterdam criteria, requiring the presence of at least two of the following: (1) oligo- or amenorrhea; (2) clinical and/or biochemical hyperandrogenism (Ferriman–Gallwey score > 8 and/or free androgen index > 4); and (3) polycystic ovarian morphology on transvaginal ultrasonography.

Exclusion criteria included pregnancy or lactation; known hypersensitivity to UC-MSCs or secretome components; current use of hormonal therapy, glucocorticoids, or pharmacological agents affecting insulin sensitivity within three months prior to enrollment; and refusal or inability to comply with study procedures. To ensure diagnostic specificity for PCOS, patients with other endocrine or metabolic disorders that may mimic PCOS were excluded, including thyroid dysfunction (hypothyroidism or hyperthyroidism), hyperprolactinemia, congenital adrenal hyperplasia, Cushing’s syndrome, androgen-secreting tumors, and uncontrolled diabetes mellitus. Active or prior infection with hepatitis A, B, or C viruses or human immunodeficiency viruses was also an exclusion criterion.

All participants underwent baseline hormonal and biochemical screening to exclude alternative causes of hyperandrogenism or ovulatory dysfunction prior to randomization.

### 2.3. Randomization and Interventions

Participants were randomly assigned in a 1:1:1:1 ratio to one of four intervention groups (*n* = 10 per group) using a computer-generated randomization sequence with concealed allocation. Simple randomization was applied without blocking or stratification. The randomization sequence was generated by an independent researcher, while participant enrollment and assignment to intervention groups were performed by the clinical team. Due to the nature of the interventions, blinding of participants and care providers was not feasible; therefore, this study was conducted as an open-label randomized controlled trial.

The metformin group received metformin extended-release (XR) at a dose of 750 mg once daily for 30 days, along with a placebo intravenous infusion of 0.9% sodium chloride and placebo nasal drops. The UC-MSC group received a single intravenous infusion of UC-MSCs at a dose of 0.3 × 10^6^ cells/kg body weight, combined with placebo oral tablets and placebo nasal drops. Participants in the secretome group received placebo oral tablets and placebo intravenous infusion, together with secretome administered as nasal drops. The combination group received both a UC-MSC intravenous infusion at the same dosage and secretome nasal drops, along with placebo oral tablets.

UC-MSC infusions were administered once under medical supervision in the hospital setting. Secretome nasal drops were administered at a total daily dose of 2 mL for 30 consecutive days, delivered equally into both nostrils using a syringe. Prior to home administration, participants received standardized instruction and demonstration by trained medical personnel. All participants received a single standardized dose of UC-MSCs (0.3 × 10^6^ cells/kg body weight) and a fixed daily dose of secretome (2 mL/day), and no dose-escalation or inter-batch comparative analyses were planned, as the primary objective of this study was to evaluate clinical metabolic responses rather than product optimization.

The intranasal route was selected for secretome administration due to its non- invasive nature, favorable safety profile, and evidence supporting systemic absorption of bioactive molecules through the nasal mucosa. The nasal epithelium is highly vascularized and enables transport of proteins, peptides, and extracellular vesicles into the systemic circulation while avoiding first-pass hepatic metabolism. Previous studies have demonstrated that intranasally administered biologics can exert systemic metabolic and immunomodulatory effects.

Although participant and care provider blinding was not feasible due to the nature of the interventions, potential bias was minimized by the use of objective metabolic and hormonal biomarkers as primary outcomes. Laboratory analyses were conducted using standardized and validated procedures, thereby reducing subjectivity in outcome assessment. Nevertheless, the absence of blinding remains a limitation, and future studies should consider incorporating blinded outcome assessment or sham-controlled designs to further reduce bias.

### 2.4. Preparation of UC-MSCs

Umbilical cord mesenchymal stem cells (UC-MSCs) were derived from umbilical cords obtained from qualified donors following written informed consent. Donors were screened and confirmed negative for HBsAg, anti-HCV, anti-HIV, TPHA, anti-CMV IgM, and anti-Toxoplasma IgM. Umbilical cord tissue was transported in sterile transport medium to a Good Manufacturing Practice (GMP)-certified facility (PT Prodia StemCell Indonesia, Jakarta, Indonesia), where UC-MSCs were isolated using the explant method and cultured in a growth medium containing Dulbecco’s Modified Eagle Medium (DMEM) and human platelet lysate. Cells were incubated at 37 °C in a humidified atmosphere with 5% CO_2_, with media changes every 3–4 days until approximately 80% confluence was reached. Cells were harvested, cryopreserved as a Master Cell Bank, and subsequently expanded to produce fresh clinical-grade products. All UC-MSC preparations fulfilled the International Society for Cellular Therapy (ISCT) minimal criteria, including plastic adherence, spindle-shaped morphology, expression of CD73, CD90, and CD105, and lack of CD34, CD45, CD14, CD19, and HLA-DR expression. Cell viability exceeded 90% at the time of administration, and all products met GMP release criteria, including sterility, endotoxin, and mycoplasma testing.

### 2.5. Preparation of UC-MSCs-Derived-Secretome

The UC-MSC-derived-secretome was obtained from the conditioned culture medium collected during the cell culture process. As UC-MSCs are adherent cells, the conditioned medium was carefully harvested without disrupting the cell layer. The collected medium was centrifuged to remove cellular debris and subsequently passed through sterile filtration to ensure product sterility. The final secretome product was aseptically packaged in sterile 3-mL syringes containing 2 mL of solution. Prior to clinical use, all secretome products underwent batch-wise quality control testing, including sterility, endotoxin, and mycoplasma assays, in accordance with GMP standards.

The UC-MSC secretome used in this study represents a complex mixture of soluble proteins, growth factors, cytokines, extracellular vesicles, and microRNAs produced by UC-MSCs during culture. In accordance with the exploratory clinical designof this study, the secretome was not further fractionated or quantitatively profiled for individual components prior to administration. Product consistency was ensured through standardized cell culture conditions, fixed collection time points, and batch-wise quality control testing.

### 2.6. Outcomes and Follow-Up

Metabolic biomarkers, including fasting plasma glucose, fasting insulin, HOMA-IR, serum C-peptide, and adiponectin, were measured at baseline and at at 1-, 3-, and 6-month follow-up. No changes to the pre-specified outcome measures were made after trial commencement. Blood samples were collected after an overnight fast. Serum insulin, C-peptide, and adiponectin concentrations were measured in duplicate using commercially available enzyme-linked immunosorbent assay (ELISA) kits (R&D Systems, Minneapolis, MN, USA) according to the manufacturer’s instructions. Fasting glucose was measured using standard enzymatic methods. HOMA-IR was calculated using the standard homeostasis model assessment formula. Assay sensitivity and intra- and inter-assay coefficients of variation were within the acceptable ranges specified by the manufacturer.

Receiver operating characteristic (ROC) curve analysis was conducted to explore the predictive performance of metabolic biomarkers with respect to treatment response at 6 months. Therapy response was defined based on clinically meaningful improvements in key metabolic parameters relative to baseline values, reflecting a favorable biological response to the intervention. The selected thresholds were determined based on changes considered clinically relevant for insulin sensitivity and metabolic function in PCOS, rather than solely on statistical significance.

### 2.7. Statistical Analysis

**Figure A1:**
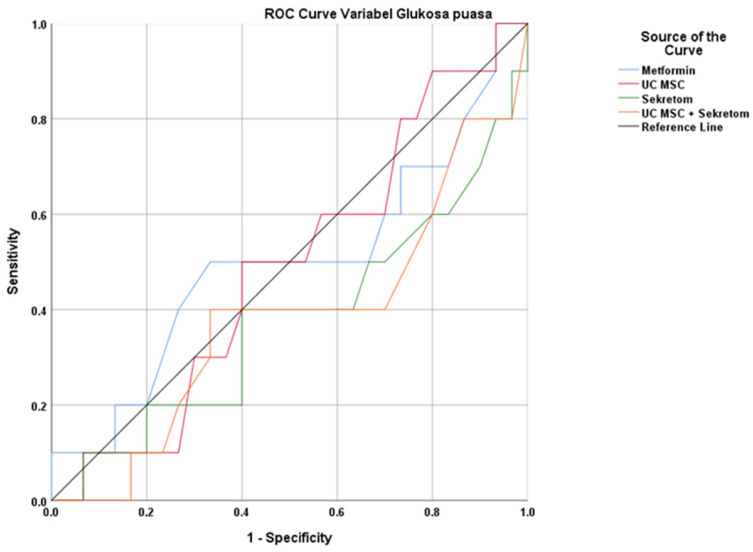

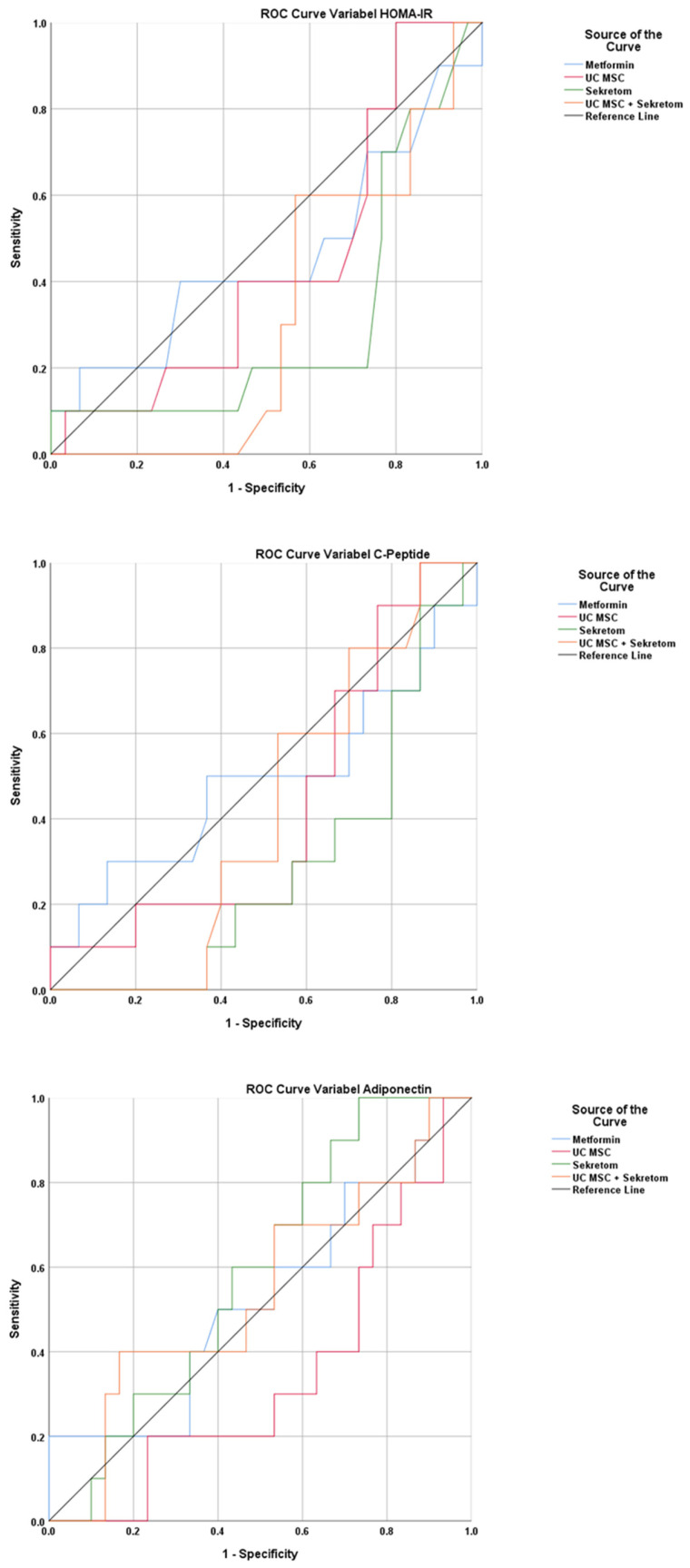
Receiver operating characteristic (ROC) curves for fasting glucose, fasting insuline, HOMA-IR, C-Peptide, and adiponectin in all treatment groups.

Statistical analyses were performed using SPSS version 27.0 (IBM Corp., Armonk, NY, USA). Data distribution was assessed using the Shapiro–Wilk test. Given the small sample size in each treatment group (*n* = 10), normality was assessed carefully using the Shapiro–Wilk test. Accordingly, repeated-measures analysis of variance (ANOVA) with Greenhouse–Geisser correction was used only for normally distributed variables, while the Friedman test was applied for non-normally distributed data. Results are therefore interpreted as exploratory, with emphasis on observed trends rather than definitive effect estimates. Within-group longitudinal changes were analyzed using repeated-measures ANOVA or the Friedman test, followed by Bonferroni-adjusted post hoc comparisons when appropriate. Between-group differences at each time point were evaluated using one-way ANOVA or Kruskal–Wallis tests, as appropriate. Receiver operating characteristic (ROC) curve analysis was and optimal cut-off values were determined using the Youden index. to assess the discriminative ability of selected biomarkers. The ROC curves are presented in Appendix A (Figure A1). A two-sided *p*-value < 0.05 was considered statistically significant. In addition to *p*-values, effect sizes and 95% confidence intervals (CIs) were reported where applicable to provide estimates of effect magnitude and clinical relevance. No interim analyses or predefined stopping guidelines were planned or conducted.

Biomarkers are reported using standardized nomenclature, and abbreviations are used consistently after the first definition.

## 3. Results

### 3.1. Baseline Characteristics

A total of 40 participants were randomized equally into four treatment groups: Metformin, UC-MSC, secretome, and UC-MSC + secretome (*n* = 10 each). All participants completed the 6-month follow-up and no protocol deviations affecting metabolic analyses were identified (Figure 1). The mean age was 29 years (range: 23–37 years), and all subjects fulfilled at least two Rotterdam diagnostic criteria for PCOS. The trial ended as planned after completion of follow-up for all participants. All randomized participants were analyzed according to the intention-to-treat principle.

At baseline, there were no statistically significant differences among the four treatment groups with respect to age, body mass index (BMI), or metabolic parameters, including fasting glucose, fasting insulin, HOMA-IR, C-peptide, and adiponectin (*p* > 0.05 for all comparisons) (see Table 1). The overall BMI distribution indicated a predominantly non-obese to overweight PCOS phenotype, with a comparable proportion of obese participants across groups. Baseline indices of insulin resistance, including fasting insulin levels and HOMA-IR, were comparable across all treatment groups, with no statistically significant differences observed (*p* > 0.05), indicating a balanced metabolic profile prior to intervention. This baseline comparability is clinically relevant given the established contribution of obesity to insulin resistance and low-grade inflammation in PCOS.

**Figure 2:**
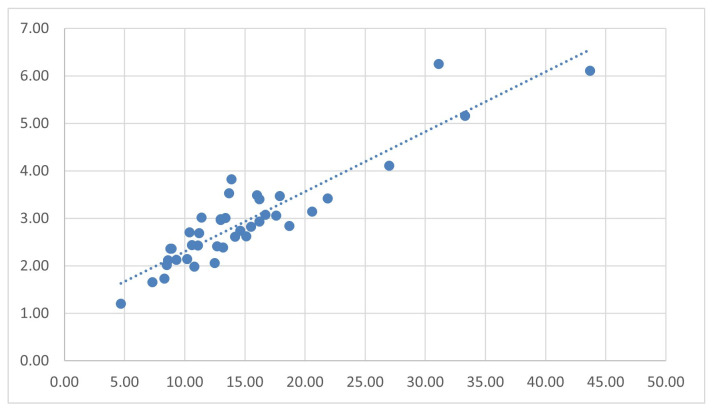
Scatterplot of fasting insulin vs. C-peptide before treatment.

In addition to descriptive analysis of individual metabolic biomarker, the relationship between baseline fasting insulin and C-peptide levels was visualized using a scatterplot, as shown in Figure 2. These parameters were selected due to their complementary roles in reflecting basal metabolic dynamics: fasting insulin reflects circulating of hyperinsulinemia, wheareas C-peptide provides a more stable indicator of endogenous pancreatic β-cell insulin secretion. This visualization illustrates the baseline metabolic compensatory profile of participants prior to UC-MSC and secretome interventions, providing a reference for evaluating longitudinal metabolic changes during follow-up.

### 3.2. Changes in Metabolic Biomarkers over Time

#### 3.2.1. C-Peptide

**Figure 3:**
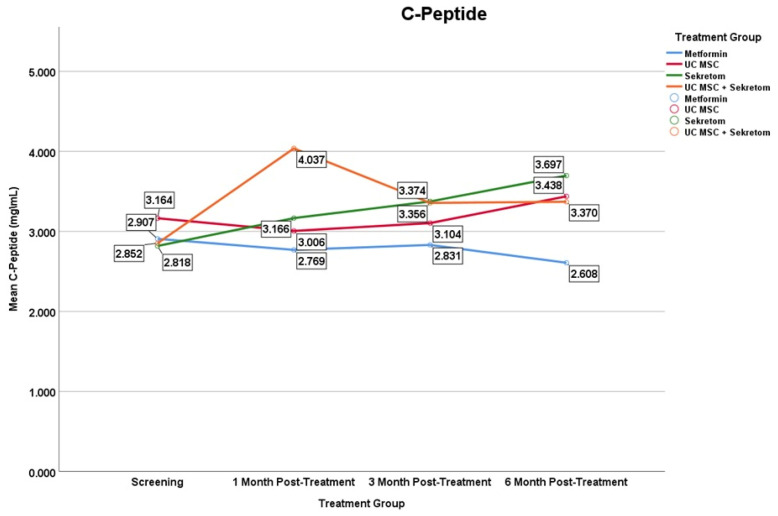
Mean serum C-Peptide levels over time in the metformin, UC-MSC, secretome, and UC-MSC + secretome groups. Figures display mean values to illustrate trends over time in small sample groups; variability is reported in the tables. Results should be interpreted as exploratory.

In C-Peptide, the Secretome group showed a significant increase at month 6 (Δ = 0.879 ± 0.69; *p* = 0.007), suggesting a possible alteration in insulin secretion dynamics; however, the absence of pharmacokinetic assessment precludes confirmation of a direct systemic effect (Figure 3). In contrast, Metformin and UC-MSC alone did not produce significant changes, while the combination of UC-MSC + secretome showed a strong initial but not sustained effect. These findings indicate a differential temporal response in endogenous insulin secretion markers across treatment groups.

#### 3.2.2. Adiponectin

**Figure 4:**
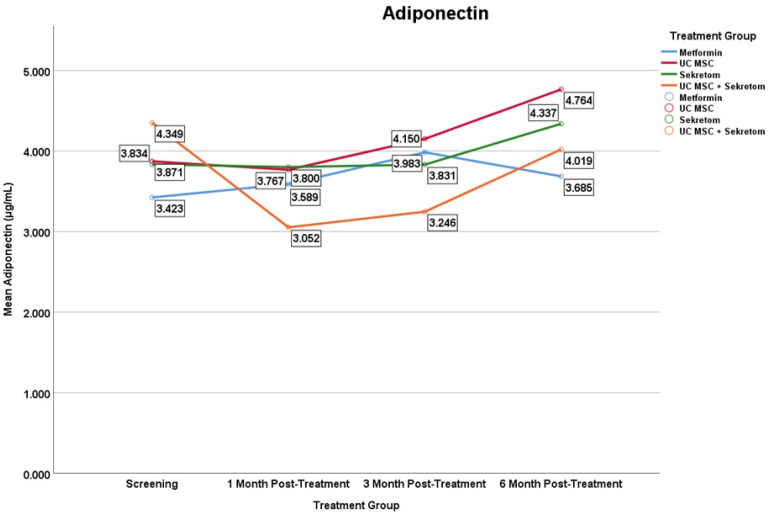
Mean serum Adiponectin levels over time in the metformin, UC-MSC, secretome, and UC-MSC + secretome groups. Figures display mean values to illustrate trends over time in small sample groups; variability is reported in the tables. Results should be interpreted as exploratory.

Regarding adiponectin levels, a significant increase in the UC-MSCs monotherapy group at month 6 compared with baseline (Δ = 0.893 ± 0.86; *p* = 0.016) (Figure 4). No significant longitudinal changes were detected in the Metformin and Secretome groups. In contrast, the combination group (UC-MSC + Secretome) group exhibited a significant decrease in adiponectin at month 1 (Δ = −1.297 ± 1.40; *p* = 0.022) and the 3rd month (Δ = −1.103 ± 0.91; *p* = 0.013). These findings suggest a possible association between UC-MSC therapy and changes in adiponectin levels; however causal inference is limited by inter-individual variability and the exploration design of the study.

#### 3.2.3. Fasting Insulin

**Figure 5:**
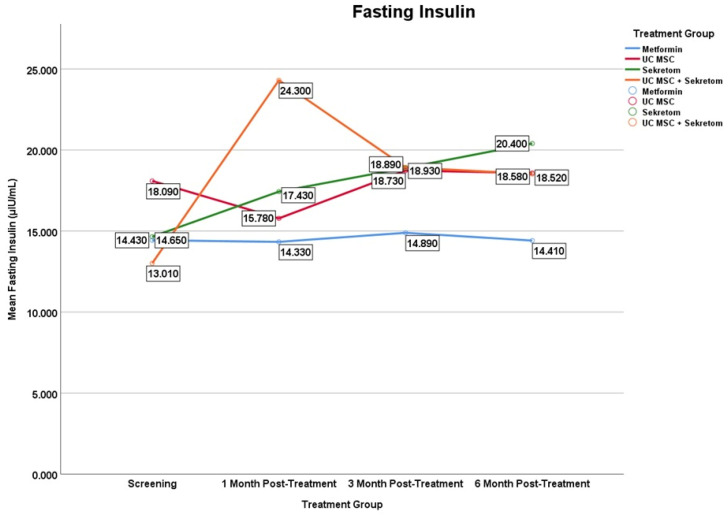
Mean serum fasting insulin levels over time in the metformin, UC-MSC, secretome, and UC-MSC + secretome groups. Figures display mean values to illustrate trends over time in small sample groups; variability is reported in the tables. Results should be interpreted as exploratory.

The secretome treatment group and the UC-MSC + secretome combination treatment group showed a significant increase in fasting insulin levels, with different time points for the onset of the effect, as shown in Figure 5. The secretome group at month 6 (Δ = 5750 ± 8.25; *p* = 0.028), while the UC-MSC + secretome combination at month 1 (11,290 ± 18.24 µIU/mL (*p* = 0.047) to month 3 (5920 ± 8.20; *p* = 0.047)). No significant longitudinal changes were observed in the metformin or UC-MSC monotherapy groups.

#### 3.2.4. Fasting Glucose

**Figure 6:**
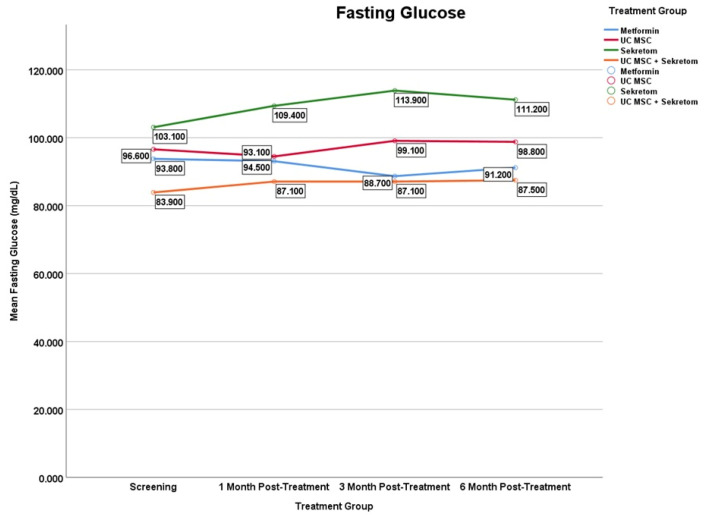
Mean serum fasting glucose levels over time in the metformin, UC-MSC, secretome, and UC-MSC + secretome groups. Figures display mean values to illustrate trends over time in small sample groups; variability is reported in the tables. Results should be interpreted as exploratory.

Changes in fasting glucose levels showed distinct temporal patterns across treatment groups ver the 6-month follow-up period (Figure 6). Among all, only the secretome group showed significant changes since the first month with an increase in the mean fasting glucose of 6300 ± 6.52 mg/dL (*p* = 0.013), which persisted at month 3 (Δ = 10,800 ± 8.55; *p* = 0.007) and remained significant until the month 6 (Δ = 8100 ± 11.23; *p* = 0.032). Meanwhile, other treatment groups such as metformin and UC MSC, even the combination of UC MSC and secretome did not show significant changes.

#### 3.2.5. HOMA-IR

**Figure 7:**
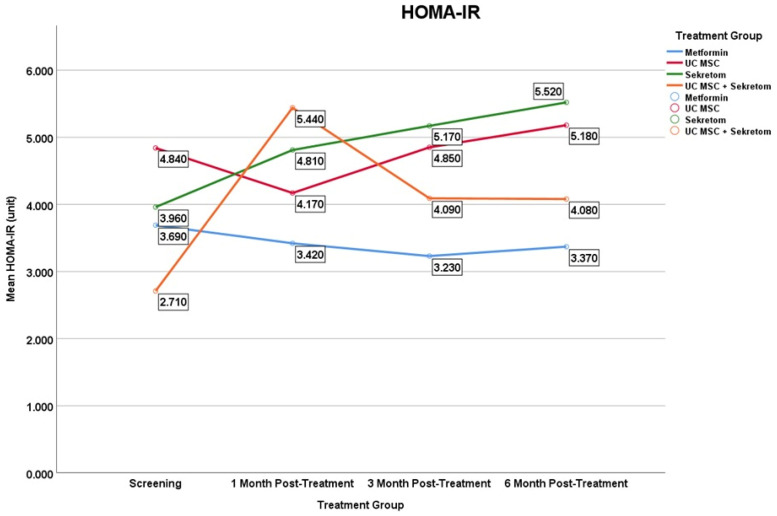
Mean serum HOMA-IR levels over time in the metformin, UC-MSC, secretome, and UC-MSC + secretome groups. Figures display mean values to illustrate trends over time in small sample groups; variability is reported in the Table 2. Results should be interpreted as exploratory.

The results showed that only the Secretome group exhibited a statistically significant increase in HOMA-IR at 6 months (Δ = 1.560 ± 2.53; *p* = 0.047), whereas no significant changes were observed in the other treatment groups (Figure 7). This phenomenon indicates a paradoxical metabolic effect of secretome on glucose regulation and insulin sensitivity in PCOS patients, which is likely related to the composition of paracrine factors in secretome that can affect insulin signaling pathways or systemic inflammation.

**Table 2:** Mean Changes (Δ ± SD) in Metabolic Biomarkers from Baseline at Each Follow-Up.

Biomarker	Group	Month 1	*p*-Value	Month 3	*p*-Value	Month 6	*p*-Value *
C-Peptide	Metformin	−0.138 ± 1.03	0.799	−0.076 ± 1.63	0.333	−0.299 ± 1.33	0.878
	UC MSC	−0.158 ± 0.72	0.508	−0.060 ± 0.99	0.878	0.274 ± 0.60	0.285
	Secretome	0.348 ± 0.59	0.093	0.556 ± 1.11	0.169	0.879 ± 0.69	0.007 *
	UC MSC + Secretome	1.185 ± 2.27	0.114	0.504 ± 1.13	0.333	0.518 ± 1.23	0.203
Adiponectin	Metformin	0.166 ± 1.15	0.799	0.560 ± 1.00	0.139	0.262 ± 1.03	0.678
	UC MSC	−0.104 ± 1.36	0.445	0.279 ± 1.14	0.959	0.893 ± 0.86	0.016 *
	Secretome	−0.034 ± 1.55	0.959	−0.003 ± 1.37	0.959	0.503 ± 1.70	0.386
	UC MSC + Secretome	−1.297 ± 1.40	0.022 *	−1.103 ± 0.91	0.013 *	−0.330 ± 2.06	0.445
Fasting Insulin	Metformin	−0.100 ± 5.69	0.959	0.460 ± 9.25	0.285	−0.020 ± 6.95	0.646
	UC MSC	−2.310 ± 6.31	0.114	0.640 ± 8.73	0.445	0.490 ± 4.78	0.878
	Secretome	2.780 ± 4.36	0.185	4.240 ± 8.77	0.114	5.750 ± 8.25	0.028 *
	UC MSC + Secretome	11.290 ± 18.24	0.047 *	5.920 ± 8.20	0.047 *	5.510 ± 11.02	0.241
Fasting Glucose	Metformin	−0.700 ± 5.91	0.959	−5.100 ± 23.47	0.799	−2.600 ± 15.92	0.959
	UC MSC	−2.100 ± 9.33	0.386	2.500 ± 6.69	0.192	2.200 ± 7.83	0.758
	Secretome	6.300 ± 6.52	0.013 *	10.800 ± 8.55	0.007 *	8.100 ± 11.23	0.032 *
	UC MSC + Secretome	3.200 ± 13.11	0.61	3.200 ± 7.05	0.213	3.600 ± 10.05	0.22
HOMA-IR	Metformin	−0.270 ± 2.02	0.959	−0.460 ± 3.60	0.575	−0.320 ± 2.76	0.683
	UC MSC	−0.670 ± 2.26	0.201	0.010 ± 2.76	0.359	0.340 ± 1.15	0.441
	Secretome	0.850 ± 1.25	0.058	1.210 ± 2.66	0.114	1.560 ± 2.53	0.047 *
	UC MSC + Secretome	2.730 ± 4.65	0.059	1.380 ± 2.02	0.053	1.370 ± 2.69	0.241

* Values are mean ± SD change from baseline (Δ). *p*-values indicate statistical significance at *p* < 0.05.

Significant changes in C-peptide were observed only in the secretome group at month 6 (*p*-value = 0.007). In adiponectin, changes occurred in the administration of UC-MSC group at month 6 month (*p*-value = 0.016), and the combination group showed significant changes at month 1 (*p*-value = 0.022) and month 3 (*p*-value = 0.013). Fasting insulin levels increased significantly in the secretome group at month 6 (*p* = 0.028), and in the UC-MSC + secretome combination group at month 1 (*p* = 0.047) and month 3 (*p*-value = 0.047). Meanwhile, fasting glucose in PCOS significantly changed with secretome administration in the 1st month (*p*-value = 0.013), month 3 (*p*-value = 0.007), and month 6 (*p*-value—0.032). Similarly HOMA-IR, there was a significant change only with secretome administration at month 6 (*p*-value = 0.047).

#### 3.2.6. Clinical Outcomes in PCOS Patients

Data are presented as n (%) or mean ± SD. ↓ indicates reduction from baseline to month 6. Due to the exploratory nature of this study and the limited sample size per group, clinical outcomes were summarized descriptively without formal between-group hypothesis testing. Categorical outcomes are presented as proportions, while continuous variables are expressed as mean ± standard deviation to illustrate temporal trends.

Based on Table 3, clinical responses after six months of therapy varied between treatment groups. Improvement in menstrual cycle regularity was most pronounced in the metformin group (80%), followed by the UC-MSC + secretome group (70%), secretome (60%), and UC-MSC (50%). Successful ovulation was recorded in 50% of patients receiving Metformin and the UC-MSC + secretome combination therapy, while a higher proportion of ovulations was found in the UC-MSC (60%) and secretome (70%) groups. Pregnancy outcomes differed between treatment groups. The highest pregnancy rate was found in the UC-MSC group (40%), all of which occurred after six months of therapy. The UC-MSC + secretome group showed a pregnancy rate of 30%, with most cases still under observation. Meanwhile, the Metformin and secretome groups showed pregnancy rates of 20% and 10%, respectively. Ovarian morphology evaluation revealed a decrease in mean ovarian volume from baseline to month 6 in all treatment groups. The decrease in ovarian volume appeared more consistent in the Metformin and UC-MSC groups, while the secretome and combination therapy groups showed milder or fluctuating changes during the observation period. No serious adverse events related to UC-MSC or secretome administration were observed during the 6-month follow-up period.

All figures and tables are presented with clearly defined units of measurement, and longitudinal biomarker changes across study time points are summarized within the main tables and figures. A downward arrow (↓) indicates a decrease.

## 4. Discussion

The prevalence of lean PCOS with insulin resistance in this study was 6 out of 40 patients (15%). Although this figure is lower compared to the prevalence of insulin resistance in PCOS patients with obesity, which can reach 35–80% [23]. The present findings of this study confirm that insulin resistance is not only limited to the obese individuals but may also occur in patients with normal BMI (non-obese). This suggests that in addition to being overweight, there are other factors such as genetic predisposition, impaired mitochondrial function, and abnormal regulation of insulin receptor signaling that can contribute to the pathogenesis of insulin resistance in lean PCOS patients [19,24,25]. This phenomenon is supported by several studies that have been conducted by several previous researchers who reported that around 6–22% of normal-weight PCOS patients showed significant insulin resistance based on metabolic tests [26,27,28], and other studies have also stated that metabolic abnormalities can be found in lean women with PCOS, even though they do not show symptoms of classic metabolic syndrome [29].

Furthermore, folliculogenesis in PCOS patients can occur optimally when fasting insulin levels are increased [30]. Data show that although the HOMA-IR value or the ratio of fasting glucose to fasting insulin (ratio) indicates increased insulin resistance, stem cell therapy actually triggers an increase in fasting insulin secretion [31]. This increase is thought to be a compensatory mechanism to maintain the folliculogenesis process [32]. Thus, stem cells can support folliculogenesis by stimulating fasting insulin secretion, while metformin works by the opposite mechanism, namely reducing insulin resistance to improve ovarian function [20].

Metabolic parameters showed an increase in adiponectin, indicating improved insulin sensitivity and reduced oxidative stress. Adiponectin is known to have anti-inflammatory effects and enhance fatty acid oxidation and glucose uptake by skeletal muscle [14]. Hormonally, there was a significant increase in FSH and AMH levels in the combination group. Increased FSH suggests stimulation of folliculogenesis, while increased AMH, in the context of inflammatory and metabolic improvements, may reflect improved granulosa cell function and oocyte quality [19]. This suggests that combination therapy is not only symptomatic but also restorative for ovarian function.

Meanwhile, the metformin group, a conventional therapy for PCOS patients, demonstrated a more limited effect, with no significant changes in most inflammatory and hormonal parameters (*p* > 0.05). This finding aligns with previous studies that suggest that although metformin is effective in lowering blood glucose and improving HOMA-IR, its effects on inflammatory cytokines and hormonal parameters are variable and limited. The effectiveness of metformin in PCOS is generally more focused on glucose regulation and does not directly suppress inflammatory cytokines such as IL-1β [33]. While the secretome and single UC-MSC groups showed more fluctuating results, they still showed improvements in fasting glucose, free testosterone, and the LH/FSH ratio. This fluctuating effect may be due to individual variation in biological response to single therapy, or because the duration and dose of therapy have not yet reached the optimal threshold for significant impact [20].

Metabolic biomarkers are key in PCOS patients with insulin resistance. AUC analysis results indicate that, in general, the classification ability of fasting glucose, fasting insulin, and C-peptide variables is very low (AUC < 0.5), and they do not have adequate classification value in assessing short- or medium-term therapy response in PCOS patients with insulin resistance. This is due to several factors, including insufficient therapy duration to significantly alter the metabolic profile, high inter-individual biological variability, and so on. However, the fasting glucose/fasting insulin ratio and adiponectin show a more promising response, although not yet significant.

Epigenetic changes that occur are: 1. The effect of DNA methylation on PCOS is characterized by hypomethylation of LHCGR (functioning in ovulation and luteinization), increased expression, resulting in chronic anovulatory disease, resulting in polycystic ovaries. Meanwhile, hypermethylation of FSHR in granulosa cells (animal studies) decreases expression. Similarly, metabolic decline due to INSR hypermethylation, C-peptide (a marker of endogenous insulin), increases, leading to insulin resistance. 2. Histone modification in PCOS occurs in granulosa and cumulus cells, beginning with the removal of active groups/acetyl groups (deacetylation), thus inhibiting the transcription process. LH HCG signals activate HDAC2 via CK2Alpha, causing global H3K27 deacetylation in granulosa cells, a process essential for ovulation-related chromatin remodeling. HDAC deacetylation inhibits transcription. Deacetylation of histone H3 and methylation of H3K9 increases in PCOS which has an impact on reducing the expression of CYP19A1 (the gene encoding/coded aromatase protein) suppressing aromatase thereby increasing androgen hormones by reducing androgen aromatization into estradiol [34,35,36]. 3. Control mechanism of non-coding RNA (microRNA/miRNA), dysregulation of inflammatory and anti-inflammatory cytokines, and resulting in metabolic dysregulation that impacts normal menstrual cycle disorders, reproductive disorders and decreased quality of life, so treatment/medication is needed to restore the process to balance/regulated, reduce the risk of long-term complications and inhibit the risk of degenerative diseases. This is owned by Umbilical Cord-Mesenchymal Stem Cells (UC-MSC) which contain genetic materials (miRNA), growth factors, inflammatory and anti-inflammatory cytokines and other normal intracellular proteins. UC-MSCs are known to produce bioactive factors that can modulate gene expression through epigenetic mechanisms, such as DNA methylation and miRNA regulation, with the effect of improving insulin sensitivity and reducing androgen levels [19,20,37,38,39].

PCOS patients are known to experience infertility due to poorly functioning ovaries. This study demonstrated that all four therapy groups: Metformin, Umbilical Cord Mesenchymal Stem Cells (UC-MSCs), secretome, and the combination (UC-MSCs + secretome) responded positively to the restoration of ovarian function. These findings indicate that stem cell-based therapy, particularly the combination of UC-MSCs and secretome, has the potential to provide a synergistic effect in stimulating follicular maturation and oocyte release. Possible underlying mechanisms include modulation of the ovarian microenvironment (inflammation, vascularization) and stimulation of granulosa cells through paracrine factors released by MSCs/secretome. MSCs are generally able to improve ovarian function through various mechanisms such as stimulating angiogenesis, reducing granulosa cell apoptosis, and promoting follicular regeneration [40]. The secretome has been identified as containing bioactive components (growth factors, extracellular vesicles) that modulate tissue regeneration and microvessel formation in the reproductive organs [41]. However, when looking at the clinical pregnancy aspect (pregnancy progressing to live birth), the pattern emerged slightly differently, with the single UC-MSC group showing the highest live birth success compared to the metformin, secretome, and combination (UC-MSC and secretome) groups. This indicates that although ovulation is a crucial step, successful embryo implantation and pregnancy continuation appear to be more dependent on endometrial stability, immune balance, and local angiogenic conditions, which may be better maintained with single UC-MSC therapy than with the combination. Recent literature suggests that MSCs are able to modulate the immune system (including macrophages and regulatory T cells) and reduce local inflammation, factors that are crucial for implantation and early embryo growth [42]. Spontaneous abortion rates were relatively low in all groups. The absence of abortion in the UC-MSC group supports the hypothesis that MSCs have protective immunomodulatory and anti-inflammatory effects: for example, through increased secretion of anti-inflammatory cytokines such as Interleukin 10 (IL-10) and Transforming Growth Factor Beta (TGF-β), and suppression of excessive Th1 inflammatory responses, which are often associated with implantation failure or early pregnancy loss. For example, the incorporation of MSCs into scaffolds has been shown to increase IL-10 expression and reduce pro-inflammatory factors such as IL-1β and IL-6 in a damaged endometrium model [43]. These results suggest that UC-MSC-based therapy has great potential in the reproductive context (particularly for the restoration of ovulation and pregnancy continuation) compared with conventional therapy (Metformin) or secretome alone. The combination of UC-MSC + Secretome appears superior in increasing ovulation but has not been accompanied by a proportional increase in subsequent pregnancy success. This confirms that in developing regenerative therapies for patients with ovulatory disorders or infertility, not only are ovulation induction factors important, but also microenvironmental factors in the endometrium, angiogenesis, and local immunology need to be considered to optimize pregnancy outcomes.

In this study, the secretome was administered via the intranasal route. Although intranasal delivery is often associated with central nervous system targeting, substantial evidence supports the ability of this route to facilitate systemic absorption of bioactive molecules through the highly vascularized nasal mucosa while avoiding first pass hepatic metabolism. The nasal epithelium enables transport of proteins, peptides, and other biomacromolecules into the circulation, providing a non-invasive route for systemic action and improved bioavailability compared to oral administration [44]. Preclinical studies have also shown that extracellular vesicles delivered intranasally can be taken up and distributed within tissues, including brain regions, indicating that intranasal delivery has potential to distribute bioactive components beyond the site of administration [45]. However, the absence of direct pharmacokinetic or biodistribution measurements in this clinical trial precludes definitive conclusions about systemic exposure of secretome factors following intranasal administration.

In terms of clinical outcomes, the results of this study indicate that each treatment modality produces distinct clinical response patterns in PCOS patients with insulin resistance. Metformin demonstrated superiority in improving menstrual cycle regularity, which is consistent with its mechanism of action in increasing insulin sensitivity and reducing hyperinsulinemia, thus improving regulation of the hypothalamic–pituitary–ovarian axis. Conversely, the highest proportion of ovulations was found in the Secretome group. This finding supports the hypothesis that paracrine factors contained in Secretome, including growth factors and anti-inflammatory cytokines, play a role in improving the ovarian microenvironment and supporting follicle maturation.

The UC-MSC group demonstrated superiority in pregnancy outcomes and the most consistent reduction in ovarian volume. This indicates that UC-MSCs not only contribute to ameliorating the chronic inflammation and oxidative stress underlying PCOS but also play a role in ovarian tissue remodeling and the restoration of medium- to long-term reproductive function. The combination therapy of UC-MSC + secretome showed promising results, but with more heterogeneous responses. This indicates the complexity of the biological interactions between stem cells and paracrine mediators, which are likely influenced by the dose, timing of administration, and the relatively limited observation period.

Clinically, these findings emphasize that PCOS treatment approaches should be personalized based on the primary outcome target, whether menstrual cycle improvement, ovulation induction, or pregnancy achievement. From a clinical perspective, these findings suggest that UC-MSC-based therapies may offer a novel adjunctive approach for selected patients with PCOS, particularly those with prominent metabolic dysfunction or suboptimal response to conventional treatments. Although standardized dosing regimens and optimal timing of administration have not yet been established, the observed metabolic and reproductive responses indicate that individualized treatment strategies may be necessary. Patient selection based on metabolic phenotype, insulin resistance severity, and reproductive goals may be critical to maximize therapeutic benefit. Importantly, these results should be interpreted as preliminary, and translation into routine clinical practice will require larger, blinded clinical trials with standardized dosing protocols and long-term safety evaluation.

Although this study demonstrates distinct metabolic and reproductive effects of UC-MSC and secretome-based therapies, the underlying molecular mechanisms were not directly assessed. Based on existing evidence, these effects are likely mediated through paracrine signaling, epigenetic regulation, and immunomodulation, including alterations in inflammatory cytokine profiles, mitochondrial function, and microRNA-mediated gene regulation. Future studies should incorporate mechanistic assays such as circulating cytokine profiling, mitochondrial metabolism markers, epigenetic analyses (including DNA methylation and histone modification), and extracellular vesicle characterization to directly elucidate the pathways through which UC-MSCs and their secretome modulate insulin sensitivity and ovarian function in PCOS. Recent molecular studies further support the relevance of targeting metabolic and mitochondrial dysfunction in PCOS. Vale-Fernandes et al. (2025) demonstrated that women with PCOS exhibit elevated anti-Müllerian hormone levels alongside impaired glycolytic activity and mitochondrial dysfunction in follicular fluid, which were associated with compromised oocyte maturation and fertilization potential [46]. These findings reinforce the central role of metabolic and mitochondrial health in ovarian function and provide mechanistic context for the observed metabolic improvements following UC-MSC and secretome-based therapies in the present study. In parallel, emerging evidence highlights the contribution of cellular senescence to PCOS pathophysiology, linking metabolic stress, chronic inflammation, and ovarian dysfunction. It has been emphasized that targeting senescence-associated pathways may represent a novel therapeutic strategy in PCOS. Collectively, these insights support the hypothesis that UC-MSC-based therapies may exert their effects, at least in part, through modulation of mitochondrial function, metabolic homeostasis, and senescence-related mechanisms [47].

Inter-individual variability in metabolic and reproductive responses was observed across all treatment groups, reflecting the heterogeneous nature of PCOS. Differences in baseline characteristics, including body mass index, degree of insulin resistance, and metabolic phenotype (lean versus overweight PCOS), are likely contributors to the variability in treatment response. Although subgroup analyses based on BMI or insulin resistance severity would be valuable for identifying responder profiles, the present study was not statistically powered to perform such analyses. Future studies with larger sample sizes should incorporate stratified or multivariate analyses to better characterize predictors of response and optimize patient selection for UC-MSC-based therapies.

Although reproductive outcomes such as ovulation rate, pregnancy rate, and live birth rate represent clinically meaningful endpoints in PCOS, these outcomes were not systematically assessed in the present study. This trial was designed as an exploratory pilot study focusing primarily on metabolic and insulin-related biomarkers as early indicators of therapeutic response.

Nevertheless, improvements in insulin sensitivity and adiponectin observed in this study may have important implications for reproductive function, given the established link between metabolic dysregulation and ovulatory dysfunction in PCOS. Future adequately powered trials with longer follow-up should incorporate reproductive outcomes as predefined endpoints and apply multivariate analyses to adjust for potential confounders, including age, body mass index, baseline insulin resistance, and concomitant fertility treatments.

## 5. Strengths and Limitations

Strengths of this study include its randomized controlled design, multi-time-point metabolic biomarker assessment, and direct comparison of UC-MSC monotherapy, secretome therapy, and combination therapy. Several limitations should be acknowledged. First, the relatively small sample size limits statistical power and the ability to perform robust subgroup analyses, including stratification by body mass index or severity of insulin resistance. Second, the open-label design may introduce performance and assessment bias, although objective biochemical endpoints were used to minimize subjectivity. Third, potential confounding factors such as lifestyle modification, dietary intake, and physical activity were not fully controlled and may have influenced metabolic outcomes. Fourth, the follow-up duration was limited to six months, which may not capture long-term sustainability or delayed adverse effects.

Future studies should incorporate larger, adequately powered cohorts, blinded outcome assessment or sham-controlled designs, standardized lifestyle monitoring, and extended follow-up periods to address these limitations and strengthen causal inference. In addition, the absence of pharmacokinetic and pharmacodynamic evaluation of intranasally administered secretome. Consequently, systemic exposure and bioavailability of secretome-derived bioactive factors cannot be confirmed, and the possibility of limited penetration into the bloodstream cannot be excluded. Furthermore, although comparator groups were included, the lack of a placebo-controlled arm specifically designed to assess secretome exposure limits causal interpretation of the observed metabolic effects, which may partly reflect inter-individual variability rather than treatment-specific activity.

## 6. Conclusions

This randomized controlled study demonstrates that UC-MSC and secretome-based therapies produce distinct metabolic effects in women with PCOS and insulin resistance. UC-MSCs monotherapy was associated with the most favourable metabolic profile, including a delayed but significant increase in adiponectin without adverse changes in glucose or insulin-related parameters. In contrast, secretome therapy was characterized by increases in fasting glucose, insulin, C-peptide, and HOMA-IR, suggesting compensatory hyperinsulinemia rather than improved insulin sensitivity. Combination therapy was associated with early but transient metabolic alterations, including temporary reductions in adiponectin, without sustained benefit at month 6. Overall, UC-MSC monotherapy showed more consistent metabolic trends in this cohort; however, the role of combination therapy in PCOS requires further investigation in placebo-controlled studies with pharmacokinetic and pharmacodynamic evaluation.

## Figures and Tables

**Table 1:** Baseline Serum Concentrations of Fasting Glucose, Fasting Insulin, HOMA-IR, C-Peptide, and Adiponectin Across Treatment Groups Prior to Intervention.

Biomarker	Metformin (*n* = 10)	UC-MSC (*n* = 10)	Secretome (*n* = 10)	UC-MSC + Secretome (*n* = 10)	*p*-Value *
C-Peptide	2.907 ± 1.63	3.164 ± 1.18	2.818 ± 0.52	2.852 ± 0.55	0.964
Adiponectin	3.423 ± 1.11	3.871 ± 1.40	3.834 ± 1.16	4.349 ± 1.48	0.891
Fasting Insulin	14.430 ± 9.95	18.090 ± 10.33	14.650 ± 3.99	13.010 ± 3.27	0.657
Fasting glucose	93.800 ± 23.36	96.600 ± 34.81	103.100 ± 47.06	83.900 ± 6.45	0.615
HOMA-IR	3.690 ± 3.49	4.840 ± 5.39	3.960 ± 3.08	2.710 ± 0.73	0.703

* Values are presented as mean ± standard deviation (SD) for descriptive purposes. Given the small sample size per group (*n* = 10), these values should be interpreted cautiously and in conjunction with non-parametric statistical analyses where appropriate. *p*-value calculated using one-way ANOVA comparing all four groups.

**Table 3:** Clinical Outcomes After 6 Months of Therapy in Patients with Polycystic Ovary Syndrome.

Outcome	Metformin (*n* = 10)	UC-MSC (*n* = 10)	Secretome (*n* = 10)	UC-MSC + Secretome (*n* = 10)
Improved menstrual cycle, n (%)	8 (80%)	5 (50%)	6 (60%)	7 (70%)
Ovulation achieved, n (%)	5 (50%)	6 (60%)	7 (70%)	5 (50%)
Pregnancy, n (%)	2 (20%)	4 (40%)	1 (10%)	3 (30%)
During observation	1	0	1	2
After 6 months therapy	1	4	0	1
Ovarian volume (mL)				
Baseline	23.1 ± 8.9	24.8 ± 9.6	11.0 ± 5.9	13.3 ± 3.6
Month 6	13.1 ± 5.4	11.9 ± 5.1	11.6 ± 5.6	9.6 ± 2.8
Trend in ovarian volume	↓ Progressive	↓ Progressive	↓ Mild	↓ Fluctuating

## Data Availability

The datasets generated and/or analysed during the current study are available from the corresponding author on reasonable request.

## Abbreviations

The following abbreviations are used in this manuscript:

AES	Androgen Excess Society
AUC	Area Under the Curve
BCAA	Branched-Chain Amino Acids
CI	Confidence Interval
DMEM	Dulbecco’s Modified Eagle Medium
ELISA	Enzyme-Linked Immunosorbent Assay
FHS	Follicle-Stimulating Hormone
FG	Fasting Glucose
FI	Fasting Insulin
HDAC	Histone Deacetylase
IR	Insulin Resistance
IL	Interleukin
MSC	Mesenchymal Stem Cell
PCOS	Polycystic Ovary Syndrome
RCT	Randomized Controlled Trial
ROC	Receiver Operating Characteristic
TGF-β	Transforming Growth Factor Beta
UC-MSC	Umbilical Cord–Derived Mesenchymal Stem Cell
XR	Extended Release
